# Evaluation of public awareness and performance toward the safe use of household disinfectants-cleaners to prevent COVID-19 in the Emirate of Abu Dhabi

**DOI:** 10.3389/fpubh.2023.1214240

**Published:** 2023-06-28

**Authors:** Nisreen Alwan, Shatha Almazrouei, Mariam Almazrouei, Jawaher Aldhaheri, Fahad Alismaili, Wissam Ghach

**Affiliations:** ^1^College of Health Sciences, Abu Dhabi University, Abu Dhabi, United Arab Emirates; ^2^Faculty of Communication, Arts and Sciences, Canadian University Dubai, Dubai, United Arab Emirates

**Keywords:** COVID-19, awareness, performance, disinfectants, household cleaners, United Arab Emirates (UAE)

## Abstract

**Introduction:**

Disinfection is one of the most effective hygienic practices that would limit the spread of the Coronavirus Disease (COVID-19) through deactivating the coronavirus on contaminated skin, supplies, and surfaces. However, the type and concentration of disinfectants should be carefully selected to avoid damaging surfaces and to limit the side effects of these chemicals on household members and users. The aim of this study is to assess the public levels of awareness and performance concerning the safe use of household cleaning products and disinfectants during the spread of COVID-19 in the Emirate of Abu Dhabi.

**Methods:**

The cross-sectional study was conducted between October and December 2021 among 750 residents of Abu Dhabi, Al Dhafrah, and Al-Ain regions. A google survey was distributed electronically for the online recruitment of the general population. Mann-Whitney and Kruskal-Wallis tests were used to determine whether significant differences exist in the levels of awareness and performance with regard to gender, region, education level, and diagnosis with COVID-19. Spearman correlation was used to test if any correlation existed between levels of awareness and performance. Kruskal-Wallis test was also used to check if significant differences exist in the mean score of performance with respect to irritation-to-poisoning symptoms.

**Results:**

The study population recorded a lower mean score of awareness (5.37 out of 12) than performance (11.75 out of 16). The majority of the study population claimed a minimum of one irritation-to-poisoning symptom during the handling of household cleaners and disinfectants. Significant differences exist in the awareness and performance mean scores among various educational levels (*P* < 0.001). The level of awareness was statistically significant with regard to infection with SARS-CoV-2 (*P* < 0.05). Also, the level of performance was significantly different between males and females (*P* < 0.001). Kruskal-Wallis test showed that the mean score of performance is statistically significant with all the studied irritation-to poisoning symptoms (*P* < 0.05).

**Conclusions:**

Awareness campaigns and training programs are recommended to address the safe use of household cleaning products and disinfectants in the United Arab Emirates (UAE).

## 1. Introduction

The Coronavirus Disease 2019 (COVID-19), caused by the novel coronavirus SARS-CoV-2, was responsible for causing the cataclysmic pandemic globally (1). This has brought the world to a standstill creating a universal huge impact. As a result, more than 500 million infected cases and six million deaths were reported throughout the world (2). SARS-CoV-2 is transmitted mainly through human-to-human and surfaces-to-human close interactions especially when the surfaces are exposed to coughing, sneezing, and aerosols from COVID patients (3). To limit the spread of SARS-CoV-2, WHO has recommended social distancing and regular practices of hygiene on the personal and household levels (4).

Disinfection is one of the hygienic practices that provide an effective way to limit the spread of COVID-19 through deactivating the coronavirus on contaminated skin, supplies, and surfaces (4). Cleaning with soapy water using mechanical processes such as brushing or scrubbing removes dirt, debris, and organic elements, but may not kill microorganisms (5). Therefore, a chemical disinfectant is required after cleaning with soap and water to kill any remaining microorganism. Ethanol, isopropanol, hydrogen peroxide, sodium hypochlorite, other chlorine-containing disinfectants, benzalkonium chloride, or quaternary ammonium compounds are all examples of chemical disinfectants (6). However, the type and concentration of disinfectants should be carefully selected to avoid damaging surfaces and to limit the side effects of these chemicals on household members and users. For example, alcohol-based hand sanitizers may cause dermal and digestive irritation to death when ingested or dermally absorbed by accident (7). Household cleaners (e.g., sodium hypochlorite) may lead to acute lung diseases and cancer when mixed with acid or amine products, respectively (8). Thus, users shall be well-educated about these chemicals for safe utilization with no unintended health risks.

Since the beginning of the pandemic, several medical centers around the world have reported accidental poisoning cases related to the misuse of disinfectants (9–11). Responding to their anxiety about being infected with SARS-CoV-2, people increased their hygiene practices (e.g., sanitizer use) and thereby increased the risk of exposure to chemical disinfectants when misused (8, 9, 12). Between 2019 and 2020, Dammam Poison Control Center in Saudi Arabia revealed that the public exposure increased from 9.8 to 20.4% and from 0.4 to 3.4% for surface disinfectants and hand sanitizers, respectively (9). Additionally, CDC recorded in its Morbidity and Mortality Weekly Report (April 2020) a sharp increase in the chemical exposures (COVID cleaners and disinfectants) in the United States of America (USA) (13). Among the reported cases, an adult woman claimed difficulty breathing, coughing, and wheezing following her smelling of a mixture of 10% bleach solution, vinegar, and hot water (13). Also, a pre-school aged child claimed dizziness, vomiting, and poor responsiveness after ingesting an unknown amount of an ethanol-based hand sanitizer (13). The investigation among the population revealed limited knowledge and consequently high-risk practices regarding the safe handling of household cleaning-disinfection products during the spread of COVID-19 in the United States (14). According to the literature, public awareness, practices, and sources of information were the interrelated variables that affected the safe use of chemical disinfectants among the populations (14–16).

In the UAE, it had been observed that people are experiencing vast growth in their awareness because of the huge number of campaigns and activities related to the coronavirus pandemic on the internet and social media platforms (1, 17). A study conducted by Baniyas et al. (1) depicted that the majority of medical students gained awareness about the pandemic from social media (high risk of inaccurate information) and a very less fraction of around 5% got information from health practitioners. Two studies had investigated COVID-related knowledge and practices (e.g., precautions and personal hygiene) among different populations in the UAE (1, 17). However, no studies assessed the public community's awareness and performance regarding the use of cleaners and disinfectants at the household level where chemicals (*e.g.*, sodium hypochlorite) could be fatal when misused.

This study evaluates the public awareness and performance of using disinfectants and household cleaners in the UAE specifically in the Emirate of Abu Dhabi. The outcome is expected to enrich the governmental authorities, healthcare professionals, and public health researchers with new evidence on the public awareness of the safe use of household chemicals and their potential implications on the public health of the communities in UAE as part of the UN sustainability goal of well-being (SDG 3).

## 2. Methods

This cross-sectional study was conducted between October and December 2021 to evaluate the public community's awareness and performance regarding the use of household cleaning products and disinfectants in the Emirate of Abu Dhabi. An online Google survey was distributed throughout email and social media platforms.

### 2.1. Population

The total number of respondents was 750 persons residing in Abu Dhabi, Al Dhafrah, and Al-Ain regions. The study population was stratified into groups based on gender, age, educational level, and prior infection with SARS-CoV-2. Individuals below 18 years old were excluded from the study.

### 2.2. Study tool

The questionnaire was adapted from the Morbidity and Mortality Weekly Report (2020)—Centers for Disease Control and Prevention (14) and validated by experts and academicians in the field. The structure of the study tool was divided into 6 sections: (1) Sociodemographic data; (2) Participants' experience with SARS-CoV-2; (3) Sources of information about preventive measures of COVID-19; (4) 9 awareness-related items with 4-point Likert scale (strongly disagree to strongly agree); (5) 9 performance-related items with Yes/No and Always-Sometimes-Never scales; and (6) 8 symptom-related items with Always-Sometimes-Never scale. Each awareness/performance item was graded on a score of 0 for an incorrect response and 1 for a correct response. For the awareness variable, a score of ≤ 3 was considered weak, between 4 and 6 was considered average, and 7 or above was considered good awareness. Likewise, for the variable performance, scores of 6 or below were considered weak, scores between 7 and 11 inclusive were considered average, and scores 12 and above were considered good performance.

### 2.3. Study analysis

The data were analyzed using the Statistical Package for the Social Sciences, version 22 (SPSS, International Business Machine Corp. IBM, Chicago, IL, USA). Percent frequency was obtained from SPSS and organized in tables to reflect the sociodemographic profile of the participants and their level of awareness and performance. The scores of awareness and performance were evaluated by descriptive analyses. Mann-Whitney test was used to determine if significant mean differences exist with regard to gender and diagnosis with COVID-19. Kruskal-Wallis test was used to check for mean differences with regard to region and education level. No comparison was made for age as the distribution was not very representative for each interval. Prior to that, normality and homogeneity of variances were tested using Kolmogorov-Smirnov and Levene's tests respectively for all variables rendering violations in these two assumptions with *P* < 0.05. Spearman correlation test was used to determine the correlation between awareness and performance of participants regarding the safe use of disinfectants-cleaners. Kruskal-Wallis test was used to investigate whether significant differences exist in awareness and performance with respect to irritation-to-poisoning symptoms. All data analysis was carried out at a significance level of <0.05.

### 2.4. Ethical considerations

The study protocol was approved by the Institutional Review Board (IRB) at the University (CoHS-21-10-32). The questionnaire included a consent form introducing the respondents to the study's purpose, benefits, confidentiality, and the absence of potential risks. In addition to that, it reinforces that participation in this study is voluntary and that the submission of the questionnaire indicates the individual's consent to participate in the study.

## 3. Results

### 3.1. Sociodemographic profile of the study population

A total of 750 participants completed the online questionnaire through email and social media outlets (Facebook, LinkedIn, and Instagram). The profile of the sample population is presented in Table 1. The Emirate of Abu Dhabi was almost equally represented by the sample population. The sex ratio (M:F) of the sample population was 1:1.12, where 683 participants (91.1 %) were between the ages of 18 and 39 years with a mean age of 27.6 (±SD = 8.39). In terms of educational level, around 70 % of the participants were well-educated (bachelor's level or higher). Looking at the medical status of the sample population, more than half of the participants (54.3 %) claimed that they have not been infected with SARS-CoV-2.

**Table 1:** Sociodemographic profile of the study population^a^.

**Variables**		**Frequency**	**Percentages (%)**
Gender	Males	353	47.10
	Females	397	52.90
	**Total**	**750**	**100.00**
Age intervals (years)	18–29	539	71.90
	30–39	144	19.20
	40–49	41	5.50
	50–64	25	3.30
	65–74	1	0.10
	75–84	0	0.00
	85 and above	0	0.00
	**Total**	**750**	**100**
Region	Abu Dhabi	264	35.20
	Al Dhafra	210	28.00
	Al-Ain	276	36.80
	**Total**	**750**	**100**
Education level	Baccalaureate or Less (High School, No Education, etc.)	218	29.10
	Bachelor	447	59.60
	Postgraduate (MSc, Ph.D., Post doctorate)	85	11.30
	**Total**	**750**	**100**
Have you been infected with SARS-CoV-2?	Yes	343	45.70
	No	407	54.30
	**Total**	**750**	**100**

^a^Values in bold refer to the total sample size.

### 3.2. Sources of information about preventive measures of COVID-19

**Figure 1:**
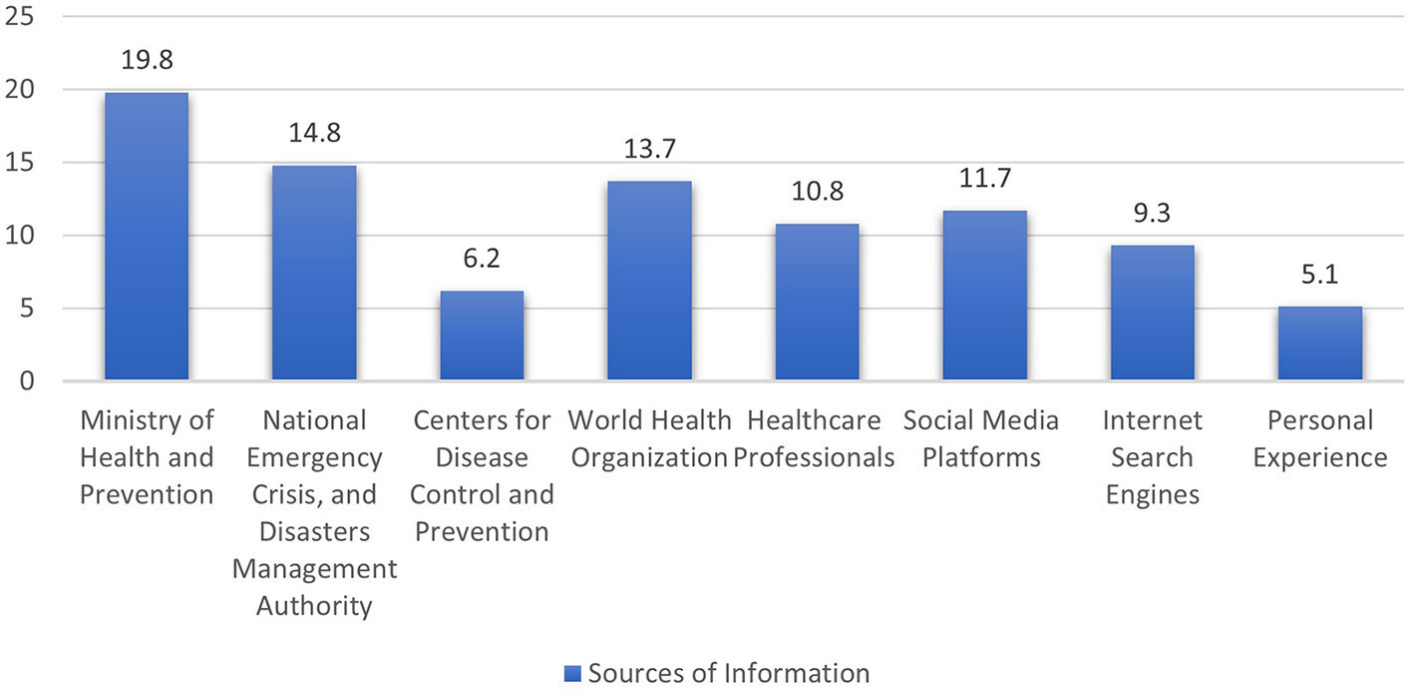
Percentage frequency of the participants' reliance on local and international sources of COVID-19 information.

Focusing on the sources of information on the preventive measures of COVID-19 (Figure 1), 65.3 % of the responses indicated that the participants obtained information from healthcare professionals, governmental agencies, international agencies, and organizations (Ministry of Health and Prevention; National Emergency Crisis and Disasters Management Authority; Centers for Disease Control and Prevention; World Health Organization; and Healthcare Professionals such as Medical Doctors, Nurses, and Public Health Specialists). On the other hand, 34.7 % of the participants' responses preferred other resources such as social media platforms, internet search engines, and personal experience.

### 3.3. Community's levels of awareness and performance

A total of nine questions were utilized to assess the community's awareness regarding the safe use of household cleaning products and disinfectants as represented in Table 2. The mean score was 5.37 out of 12 (±SD of 2.66) where only 25 % of the sample population (188 out of 750 participants) showed weak awareness of storage, personal protective equipment (PPE), and application protocols of the cleaning-disinfecting products. At the awareness level, more than 50 % of the study population did not recognize the CDC and WHO recommendations for non-mixing chlorine solution with any other substances (e.g., acid, ammonia), and keeping chlorine solution at ambient temperature (4, 18).

**Table 2:** Community's level of awareness regarding the use of household cleaning products and disinfectants^a^.

**Variables**		**Frequency**	**Percentages (%)**
Household cleaning products should be kept out of reach of children.	Somewhat agree	136	18.13
	Somewhat disagree	27	3.60
	**Strongly agree**	**574**	**76.53**
	Strongly disagree	13	1.73
Hand sanitizers should be kept out of reach of children.	Somewhat agree	209	27.87
	Somewhat disagree	40	5.33
	**Strongly agree**	**481**	**64.13**
	Strongly disagree	20	2.67
For some household cleaning products, gloves should be used during use.	Somewhat agree	169	22.53
	Somewhat disagree	35	4.67
	**Strongly agree**	**536**	**71.47**
	Strongly disagree	10	1.33
For some household cleaning products, eye protection should be used during use.	Somewhat agree	245	32.67
	Somewhat disagree	47	6.27
	**Strongly agree**	**435**	**58.00**
	Strongly disagree	23	3.07
Hands should be washed with soap and water after using household cleaning products.	Somewhat agree	155	20.67
	Somewhat disagree	25	3.33
	**Strongly agree**	**556**	**74.13**
	Strongly disagree	14	1.87
Good ventilation (airflow) is needed when using cleaning chemicals.	Somewhat agree	159	21.20
	Somewhat disagree	20	2.67
	**Strongly agree**	**556**	**74.13**
	Strongly disagree	15	2.00
Bleach should be mixed with ammonia.	Somewhat agree	109	14.53
	Somewhat disagree	187	24.93
	Strongly agree	90	12.00
	**Strongly disagree**	**364**	**48.53**
Bleach should be mixed with vinegar.	Somewhat agree	109	14.53
	Somewhat disagree	193	25.73
	Strongly agree	89	11.87
	**Strongly disagree**	**359**	**47.87**
When making a dilute bleach solution, only room temperature water should be used.	Somewhat agree	218	29.07
	Somewhat disagree	152	20.27
	**Strongly agree**	**169**	**22.53**
	Strongly disagree	211	28.13
Awareness level	Weak	188	25.07
	Moderate	231	30.80
	Strong	331	44.13

^a^Values in bold show correct responses.

A total of nine questions were utilized to assess the community's performance regarding the safe use of household cleaning products and disinfectants (Table 3). The mean score was 11.75 out of 16 (±SD = 3.16) where only 9 % of the sample population (67 out of 750 participants) showed weak performance during the use of cleaning products and disinfectants in household settings. At the performance level, more than 50 % of the study population recorded low adherence to the WHO and CDC guidelines for never inhaling the vapor of household cleaners during the hygiene process; washing vegetables, fruits, and skin with household cleaners (4, 19, 20).

**Table 3:** Community's level of performance regarding the use of household cleaning products and disinfectants^a^.

**Variables**		**Frequency**	**Percentages (%)**
Have you increased the frequency of home cleaning during COVID-19?	No	90	12.00
	**Yes**	**660**	**88.00**
Did you wash vegetables, fruits, or other food products with bleach?	**Never**	**410**	**54.67**
	Sometimes	113	15.07
	Always	227	30.27
Have you used household cleaner to clean or disinfect hands or bare skin?	**Never**	**364**	**48.53**
	Sometimes	179	23.87
	Always	207	27.60
Did you mist the body with cleaning spray or alcohol spray after being in public places?	Never	202	26.93
	Sometimes	208	27.73
	**Always**	**340**	**45.33**
Have you inhaled the vapor of household cleaners like bleach?	**Never**	**328**	**43.73**
	Sometimes	269	35.87
	Always	153	20.40
Did you drink or gargle soapy water?	**Never**	**593**	**79.07**
	Sometimes	89	11.87
	Always	68	9.07
Did you drink or gargle a household cleaner?	**Never**	**601**	**80.13**
	Sometimes	87	11.60
	Always	62	8.27
Did you drink or gargle a diluted bleach solution?	**Never**	**598**	**79.73**
	Sometimes	84	11.20
	Always	68	9.07
Did you follow the guidelines indicated on the bleach/disinfectant bottle?	No	95	12.67
	**Yes**	**655**	**87.33**
Performance level	Weak	67	8.93
	Moderate	240	32.00
	Strong	443	59.07

^a^Values in bold show correct responses.

The Spearman correlation test showed that the community awareness and performance were weakly correlated (rho = 0.251, *p* < 0.001).

### 3.4. Association of awareness and performance with the participants' sociodemographics

The mean scores of awareness and performance levels per sociodemographic variable and the total mean score of awareness and performance are shown in Table 4. Results of the Mann-Whitney test showed no significant differences in the awareness level between males and females (*P* = 0.303 > 0.05, Table 4) although females have a higher mean awareness score than males. As per the *P-*value of the Kruskal-Wallis test, no significant differences existed in the awareness level among the three regions in the Emirate of Abu Dhabi (Table 4) with the highest mean score in Al-Ain followed by Al Dhafra then Abu Dhabi, respectively. Significant differences exist in the level of awareness among different levels of education (*P* < 0.001) with the highest mean score in participants holding post graduate degrees followed by those holding a bachelor's degree and less with those with baccalaureate or less (Table 4). According to the Mann-Whitney test, significant differences exist in the awareness level between those diagnosed with COVID-19 and those who were not (*P* = 0.024 <0.05, Table 4).

**Table 4:** Awareness and performance level mean scores classified as per socio-demographic variables.

**Variables**	**Total awareness level** ^**a**^			**Total performance level** ^**a**^		
	**Mean**	**SD**	***P-*** **value** ^b^	**Mean**	**SD**	***P-*** **value** ^b^
**Gender**						
Males	5.35	2.911	0.303	12.75	2.803	**<0.001**
Females	5.40	2.429		10.85	3.203	
**Region**						
Abu Dhabi	5.25	2.729	0.755	11.93	2.991	0.491
Al Dhafra	5.32	2.956		11.72	3.370	
Al-Ain	5.53	2.355		11.59	3.169	
**Education level**						
Baccalaureate or less (High School, illiterate, etc.)	4.65	2.703	**<0.001**	10.45	3.346	**<0.001**
Bachelor	5.6	2.635		12.42	2.940	
Postgraduate (MSc, Ph.D., Post doctorate)	6.05	2.355		11.51	2.772	
**Have you been infected with SARS-CoV-2 (Clinical diagnosis)?**						
Yes	5.57	2.695	**0.024**	11.61	3.488	0.918
No	5.21	2.633		11.86	2.864	

^a^Awareness score was calculated over 12 points. Performance score was calculated over 16 points.

^b^Values in bold show significant P-values <0.05.

On the other hand, results of the Mann-Whitney test showed significant differences in the performance level between males and females (*P* < 0.001, Table 4) with males having a higher mean performance score than females. As per the *P-*value of the Kruskal-Wallis test, no significant differences existed in the performance level among the three regions in the Emirate of Abu Dhabi (*P* = 0.491 > 0.05, Table 4) with the highest mean score in Abu Dhabi followed by Al Dhafra then Al–Ain, respectively. Significant differences exist in the level of performance among different levels of education (*P* < 0.001) with the highest mean score in participants holding a bachelor's degree followed by those holding post graduate degrees and less with those with baccalaureate or less (Table 4). According to the Mann-Whitney test, no significant differences exist in the performance level between those diagnosed with COVID-19 and those who were not (*P* = 0.918 > 0.05, Table 4).

### 3.5. Symptoms encountered during the use of household chemicals

**Figure 2:**
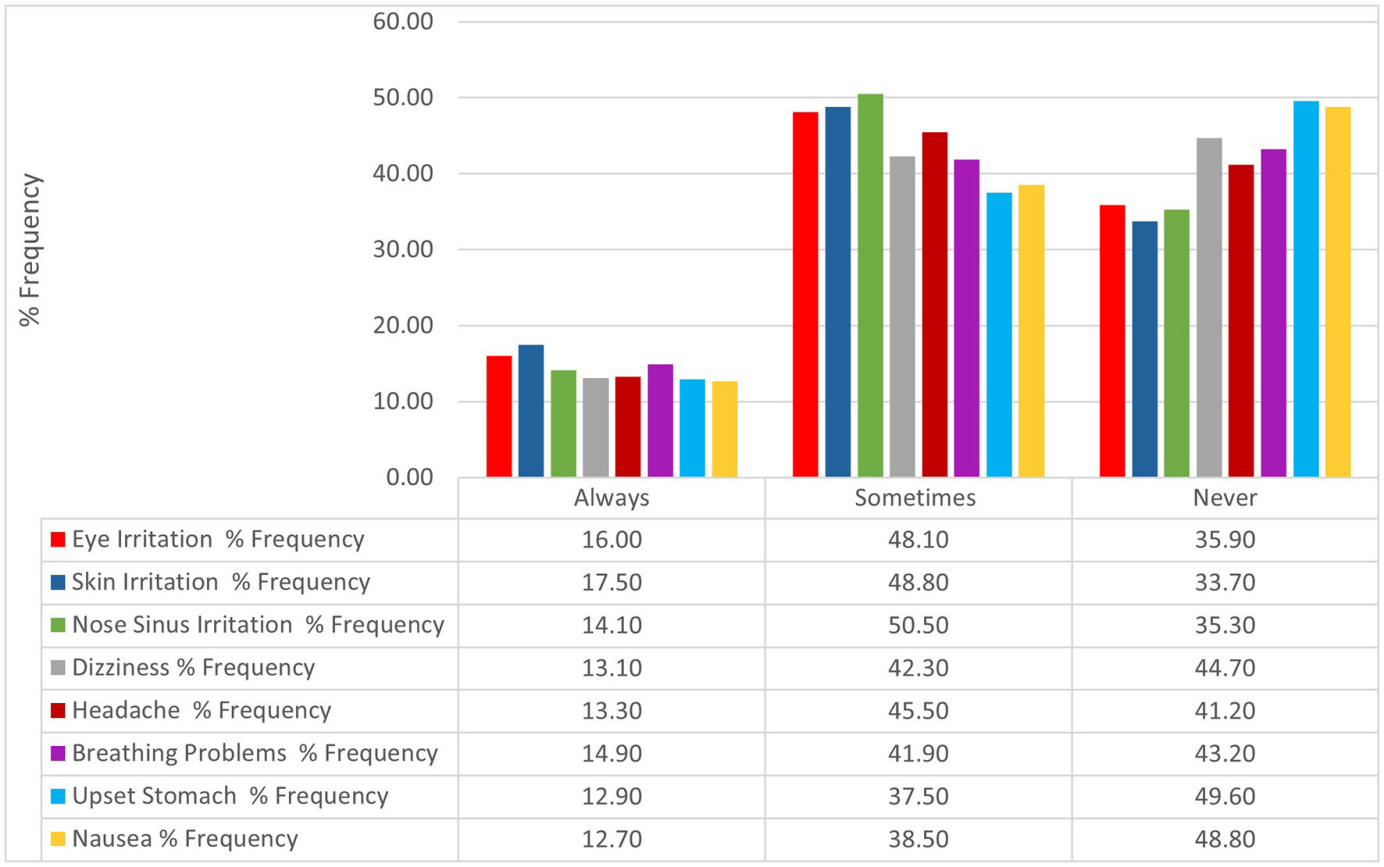
Percentage frequency of symptoms encountered by the study population.

To study the relationship between community performance and non-safe exposure to household cleaning products and disinfectants, the occurrence of irritation-to-poisoning symptoms had been investigated as shown in Figure 2. Participants who claimed no irritation-to-poisoning symptoms ranged between 33.7 % (Skin irritation) and 49.6 % (Upset stomach). However, the rest of the sample population had claimed a minimum of one irritation-to-poisoning symptom when they used household cleaners and disinfectants during COVID-19. The claimed symptoms among the sample population were comparable with the highest occurrence of skin irritation (Sometimes-to-always: 66.3 %) and the lowest occurrence of upset stomach (Sometimes-to-always: 50.4 %).

The Kruskal-Wallis test was applied to investigate significant differences in the awareness and performance level mean score among the irritation-to-poisoning symptoms. The test revealed statistical significance between the awareness and performance levels and all the studied symptoms (*P* < 0.05): Eye irritation (A & P levels: *P* < 0.001); Skin irritation (A & P levels: *P* < 0.001); Nose-sinus irritation (A level: *P* = 0.001; P level: *P* < 0.001); Dizziness (A & P levels: *P* < 0.001); Headache (A & P levels: *P* < 0.001); Breathing problems (A & P levels: *P* < 0.001); Upset stomach (A & P levels: *P* < 0.001); and Nausea (A & P levels: *P* < 0.001) (Table 5).

**Table 5:** Statistical results of awareness and performance levels as per symptoms.

**Symptoms**	**Awareness level^a^**	**Performance level^a^**
Eye irritation	*P <* 0.001	*P <* 0.001
Skin irritation	*P <* 0.001	*P <* 0.001
Nose-sinus irritation	*P =* 0.001	*P <* 0.001
Dizziness	*P <* 0.001	*P <* 0.001
Headache	*P <* 0.001	*P <* 0.001
Breathing problems	*P <* 0.001	*P <* 0.001
Upset stomach	*P <* 0.001	*P <* 0.001
Nausea	*P <* 0.001	*P <* 0.001

^a^Values in bold show significant P-values <0.05.

## 4. Discussion

This study is the first study that assessed public awareness and performance regarding the safe use of household cleaning products and disinfectants during the spread of the pandemic in the UAE. For the first time, it sheds light on the quality of the prevention measures to stop the spread of the SARS-CoV-2 virus within households in the Emirate of Abu Dhabi. An anticipated output is to provide healthcare professionals and public health scientists with new evidence on the public awareness and performance of household cleaning products and disinfectants in the Emirate of Abu Dhabi. The outcome and implications of this study are not limited to the pandemic era and may provide a database to estimate the extent of the safe use of household cleaners during day-to-day application after the pandemic. Public health initiatives may use these findings to design effective awareness campaigns using pictorial instructions, animation videos, real life-based storytelling, and technical training programs to highlight misconceptions and mishandling of cleaning products and disinfectants at the household level. Additionally, the study findings could stress on the research communities to overcome the gap of absence/limited surveys of chemical awareness through the development and validation of new tools in multiple languages for developed and developing countries, and to integrate the knowledge of chemical safety in the educational curricula at the academic institutions. In comparison with similar studies, this study population showed a higher level of awareness (moderate-to-strong) regarding the safe process of disinfection (14–16, 21). In consistent to other studies (15, 21), the awareness level was somehow low concerning the safe use of some cleaning products and disinfectants such as the acceptance of child's exposure to sanitizers which may increase the risk of children poisoning by ingesting the residue of alcohol when putting their hands in their mouths or eating food directly post disinfection with sanitizers (9, 22). Additionally, the elevation of hygiene processes along with the low awareness regarding the preparation of cleaning products may increase the risk of hazardous releases such as chloroform and chloramine, or chlorine gas when bleach is mixed with ammonia or vinegar, respectively (8, 23). Also, diluting a chlorine solution at a high temperature is not recommended where a loss of disinfection power is expected by the decomposition of hypochlorite (the active ingredient of bleach) (24).

Compared to the other Middle Eastern studies (15, 16), the study population showed a higher level of performance (moderate-to-strong) than awareness. This finding could be explained by the focus of awareness campaigns on the practical awareness of disinfectants rather than the basic and scientific knowledge (16). Similar to other published studies (14, 25), the incidence of some inappropriate practices (e.g., washing vegetables and fruits with a chlorine solution, disinfecting bare skin with chemical detergents, inhaling the smell of household cleaners, and less frequently drinking or gargling with household products) were reported in this study.

The Spearman correlation test showed a weak correlation between community awareness and performance. This finding could be explained by a presence of an third factor (attitude) who have higher influence on the participants' adherence to the safe processes than awareness variable (26). However, the attitude variable which was not investigated to prove this hypothesis.

Another important variable was the occurrence of irritation-to-poisoning symptoms among half of the study population during their use of household cleaning products and disinfectants. Specifically, headache, respiratory and dermal irritations were the main reported symptoms in comparison to the Egyptian study where multiple respiratory and dermal symptoms of chemical toxicity were commonly detected (15). The recorded symptoms were completely associated (P <0.05) with the participants' awareness and performance to confirm that the study population and their family members were somehow vulnerable to food poisoning and respiratory problems according to the low awareness and misuse of cleaning products at homes (15).

Notable variables were gender and education level, and prior infection with SARS-CoV-2 regarding the levels of awareness and performance. The gender-based finding was inconsistent with the literature where females recorded a higher awareness but lower performance scores than males (16, 27–29). The dominant role of mothers, homemakers, or cleaning staff would explain why females had a higher level of awareness concerning hygiene processes and products (30). Well-educated participants (Bachelor and higher levels) recorded higher scores of awareness and performance than less/non-educated participants. As mentioned in the literature, the higher the education level, the higher the awareness and performance levels concerning preventive measures and disinfectant use (16, 29, 31). In addition, education may play an important role in choosing reliable sources of information and avoiding any online sources with misinformation and no scientific support (32). Participants who previously experienced COVID-19 recorded a higher mean score of awareness than the participants who had never been diagnosed with COVID-19. This is in congruence with a study conducted by Aldhahri and Alghamdi (33) regarding awareness about COVID symptoms.

Several limitations are noted in this study. Findings were based on subjective analysis which may not be associated with clinical investigations. Self-responding bias is expected to affect the study finding too. The electronic data collection may weaken the randomness of the sampling especially that the lack of familiarity with the electronic devices and/or social media platforms is expected among the public community. As a result, the age distribution of the sample population was affected by the electronic procedure of data collection where most of the participants were young participants (aged 18–39) who are expected to have more accessibility to the social media platforms.

The awareness and performance levels concerning the use of household cleaning products and disinfectants were moderate-to-strong among the majority of the study population. However, public awareness was lower than their performance level along with the incidence of irritation-to-poisoning symptoms among the majority of the study population, indicating the necessity of more training programs (theoretical and practical awareness) in governmental webpages, academic institutions, private companies, and the entertainment public places of Abu Dhabi. It was also found that the regional districts of Abu Dhabi showed almost similar levels of performance and awareness. Associated variables such as gender, educational level, and prior infection with SARS-CoV-2 contributed positively to either awareness, performance, or both levels among the study population. Community-based interventions and future studies are recommended to address the above-mentioned variables in addition to the levels of awareness and performance in using cleaning products and disinfectants at the household levels in the UAE.

## Data availability statement

The raw data supporting the conclusions of this article will be made available by the authors, without undue reservation.

## Ethics statement

The studies involving human participants were reviewed and approved by the Institutional Review Board (IRB) at Abu Dhabi University (CoHS-21-10-32). The patients/participants provided their written informed consent to participate in this study.

## Author contributions

Conceptualization, formal analysis, supervision, and writing—review and editing: WG and NA. Methodology, data curation, writing—original draft preparation, and project administration: SA, MA, JA, FA, WG, and NA. All authors contributed to the article and approved the submitted version.
